# A thymidylate synthase ternary complex-specific antibody, FTS, permits functional monitoring of fluoropyrimidines dosing

**DOI:** 10.18632/oncotarget.554

**Published:** 2012-07-22

**Authors:** Kalpesh Patel, Sashidhar R. Yerram, Nilofer A. Azad, Scott E. Kern

**Affiliations:** ^1^ Department of Oncology, The Sidney Kimmel Comprehensive Cancer Center, Johns Hopkins University, Baltimore, MD, USA

**Keywords:** Ternary complex, thymidylate synthase, drug adduct, drug adduct-specific antibody, ternary complex-specific antibody, FTS

## Abstract

5-Fluorouracil (5FU) and similar fluoropyrimidines induce covalent modification of thymidylate synthase (TS) and inhibit its activity. They are often used to treat solid cancers, but drug resistance and toxicity are drawbacks. Therefore, there is an unmet need for a functional assay to quantify fluorouracil activity in tissues, so as to individually tailor dosing. It is cumbersome to separately quantify unmodified and 5FU-modified TS using currently available commercial anti-TS antibodies because they recognize both forms. We report here the first monoclonal antibody (FTS) specific to 5FU-modified TS. By immunoblot assay, the FTS antibody specifically recognizes modified TS in a dose-dependent manner in 5FU-treated cells, in cancer xenograft tissues of 5FU-treated mice, and in the murine tissues. In the same assay, the antibody is nonreactive with unmodified TS in untreated or treated cells and tissues. Speculatively, a high-throughput assay could be enabled by pairing anti-TS antibodies of two specificities, one recognizing only modified TS and another recognizing both forms, to structurally quantify the TS-inhibiting effect of fluorouracil at a cellular or tissue level without requiring prior protein separation. Such a development might aid preclinical analytic studies or make practical the individual tailoring of dosing.

## INTRODUCTION

TS catalyses the reductive methylation of 2-deoxyuridine-5-monophosphate (dUMP) to 2-deoxythymidine-5-monophosphate (dTMP) with provision of a carbon donated by 5, 10-methylene tetrahydrofolate (DMTHF) [1, 2]. dTMP is then converted to dTTP for use in DNA synthesis. As a necessary component of DNA replication, TS is an attractive target for cancer treatment.

The anti-metabolite drug 5FU, a fluoropyrimidine, and fluoropyrimidine analogues are used to inhibit TS in cancer treatment [3]. Intracellularly, 5FU is converted to active metabolites fluorodeoxyuridine (FdUMP), fluorodeoxyuridine triphosphate (FdUTP), and fluorouridine triphosphate (FUTP). FdUMP competes with dUMP and, covalently with DMTHF, binds TS to form a ternary complex (5FU-modified TS, TS-F) [1], terminating its activity. The ternary complex consists of a covalent bond between Cys198 of TS and C-6 of FdUMP and covalent bonds of the methylene group to both C-5 of FdUMP and N-5 of folate. Graded inhibition of TS results in degrees of inhibition of DNA synthesis. FdUTP can, in place of dTTP, incorporate into DNA and result in DNA damage directly by mis-incorporation or indirectly by stimulating DNA repair [4-6]. FUTP, in place of UTP, incorporates into, and damages or impairs function of, RNA [7-9].

Fluoropyrimidines are an essential component of colorectal cancer chemotherapy [10], are also used to treat other gastrointestinal cancers, breast cancer, and head and neck cancers, and are often included in combination chemotherapeutic regimens. Despite large numbers of 5FU-related clinical studies [11], there has been a little done to individually tailor fluoropyrimidine dosage for cancer therapy. The separate quantification of native unmodified TS (TS-N) and TS-F after treatment could be used to optimize dosing and tumor responses. Drake et.al, used immunoblots (IB) to quantify total TS and TS-F [12]. Quantification of total TS, TS-N and TS-F was also done using radiochemicals [13-15]. These methods are tedious at best, however.

To work toward a more facile quantification, we developed a monoclonal antibody by using TS-F as the immunizing antigen. By IB, the antibody specifically recognized TS-F from 5FU-treated cell lysates and from 5FU-treated cancer xenograft tissues. A plausible moderate-term future goal would be to quantify separately TS-N and TS-F in tissues by developing an assay that used a nonspecific anti-TS antibody and a specific anti-TS-F antibody, so as to permit clinical monitoring of fluoropyrimidine cellular activity, expressed as measured ratio of TS-F to the remaining TS-N.

## RESULTS

### Verifying the method of TS modification in vitro

**Figure 1:**
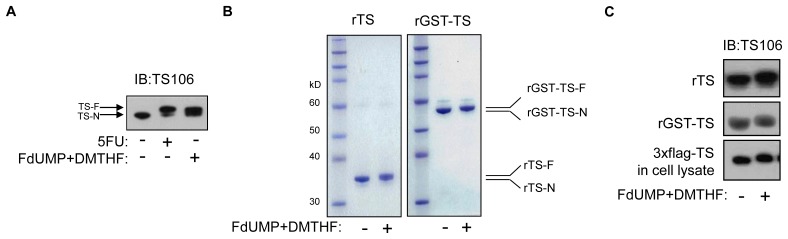
TS modification in vitro (A) RKO cells were treated with 5FU in culture, and an RKO cell lysate was modified in vitro using FdUMP and DMTHF. IB analysis was done using TS106. (B) Purified rGST-TS and rTS were modified in vitro and analyzed after separation by denaturing gel and Coomassie staining. (C) IB analysis of in vitro-modified rTS, rGST-TS, and 3xFlag-tagged TS in an RKO cell lysate, using TS106.

It is known that cellular TS-F migrates slower than TS-N in denaturing protein gels, by IB [16]. By IB using anti-TS antibody (TS106), we also observed cellular TS-F migrating slower than TS-N in the in vitro-modified RKO cell lysate (Figure 1A). Results were compared with a lysate of 5FU-treated RKO cells, in which TS-F migrates slower than TS-N.

We produced rTS and modified it in vitro to form rTS-F. In Coomassie-stained denaturing protein gels, we observed rTS-F migrating slower than un-modified rTS (rTS-N) (Figure 1B). This verified our in vitro-modification of rTS to rTS-F. We also observed in vitro modified rGST-TS-F migrating slower than unmodified rGST-TS.

By IB using TS106, we observed slower migration of rTS-F than rTS-N and, similarly, of rGST-TS-F than rGST-TS (Figure 1C). In an additional control, we observed the presence of in vitro-modified 3XFlag-tagged TS in a lysate of RKO cells transfected so as to express 3xFlag-tagged TS. After these confirmations, the purified rTS-F was used to immunize animals for antibody generation.

### Development of monoclonal antibody

We screened more than 60 hybridoma clones from mouse immunizations, but all murine clones failed to recognize rTS-F specifically. Because the chemical structures of folic acid, THF, and DMTHF are similar, and because many commercial antibodies to forms of folic acid are available, we reasoned that these antibodies might cross-react with DMTHF, which is an adduct of, and thus present in, TS-F. Immunoblotting, using various anti-FA antibodies, however failed to detect the TS-F present in 5FU-treated RKO cells (Data not shown). We continued our efforts by immunizing rats with rTS-F. From 16 rat hybridoma clones, one (FTS, rat IgG1) specifically recognized rTS-F in ELISA. We selected the FTS clone for expression and antibody purification.

### Characterization of new monoclonal antibody FTS

**Figure 2:**
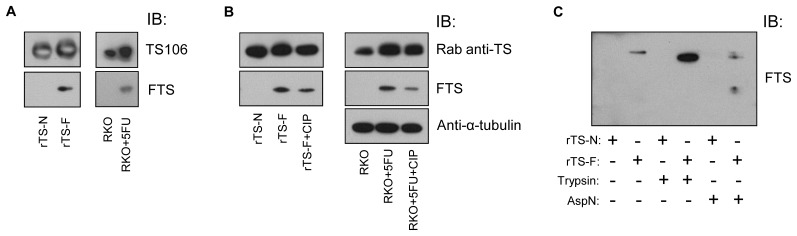
Characterization of moAb FTS (A) rTS-N, rTS-F, untreated RKO cells and 5FU-treated RKO cells were analyzed by IB using TS106 and moAb FTS. (B) rTS-F and a lysate of 5FU-treated RKO cells were treated with CIP and analyzed by IB as indicated. (B) rTS and rTS-F were digested with trypsin or AspN and analyzed by IB as indicated. Rab, rabbit.

By IB, monoclonal antibody (moAb) FTS reacted with rTS-F and not with rTS-N (Figure 2A). A commercial TS106 antibody recognized both rTS-F and rTS-N. FTS immunoreacted with TS-F from 5FU-treated RKO cells and did not detect TS-N in un-treated cells (Figure 2A). TS106 detected both forms of TS in RKO cells.

#### Phosphatase treatments of 5FU-treated RKO cell lysate and rTS-F

**Figure 3:**
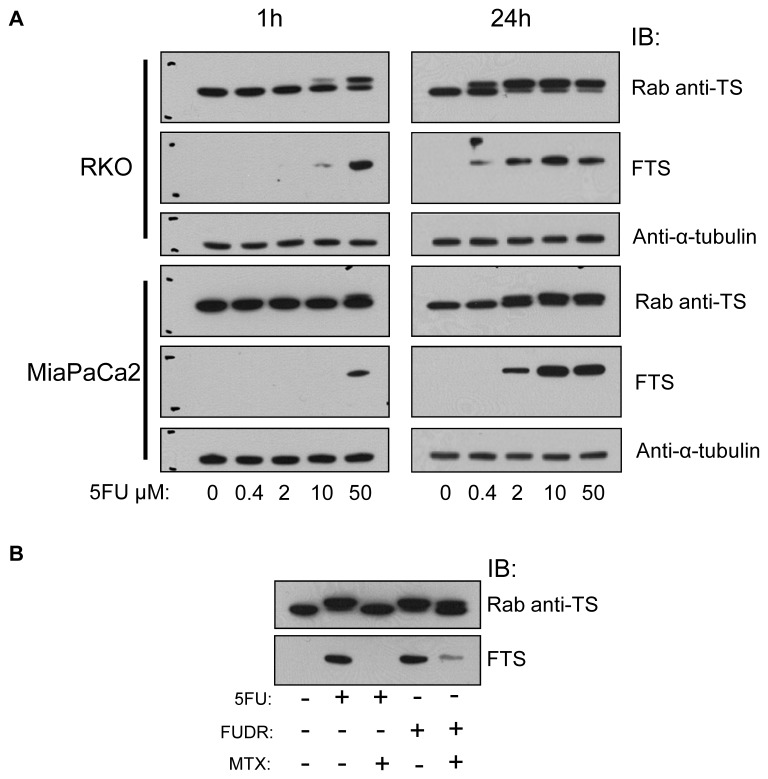
Functional monitoring of 5FU in vitro using moAb FTS (A) RKO and MiaPaCa2 cells were treated with various doses of 5FU for 1h or 24h and analyzed by IB using a rabbit anti-TS, FTS moAb, and anti-α-tubulin antibody. (B) RKO cells were treated with combinations of 5FU, FUDR and MTX, incubated for 24h, and analyzed by IB as indicated. Rab, rabbit. Rab anti-TS and FTS blots have molecular size marks, upper mark represents 40kD and lower mark represents 30kD.

CIP treatment of the lysate of 5FU-treated RKO cells abrogated the migration shift characteristic of TS-F (Figure 3A), a shift demonstrable using commercial anti-TS antibodies [17]. There was a possibility that the covalent modification might remain essentially intact after removal of the phosphate from FdUMP, despite the conversion to a more rapid migration speed. If this were the case, then one should perhaps detect the faster migration of TS-F, when using moAb FTS. Upon CIP treatment of lysates of 5FU-treated RKO cells and of purified rTS-F, however, FTS did not detect a faster migration of TS-F; rather, immunodetectable TS-F merely decreased (Figure 2B). This observation confirms the suggestion of Brody et. al, that TS-F may be unstable after phosphatase treatment. The phosphate of FdUMP is known to be ligated by four arginine residues and a serine residue in the ternary complex. Removing the phosphate might de-stabilize the ternary complex, perhaps driving the reverse reaction.

#### Trypsin and endoproteinase AspN digestions of rTS and rTS-F

Because phosphatase treatment rendered the TS modification unstable, we wondered whether TS modification were stable after proteolytic digestion. We digested rTS and rTS-F using trypsin and AspN. By IB, moAb FTS detected trimmed fragments of rTS-F (Figure 2C) after digestion with trypsin and AspN, suggesting retention of the modification in partially digested rTS-F. FTS detected in AspN digestion fragments smaller than in trypsin digestion, perhaps due to the relative resistance of rTS-F to trypsin digestion, which was described by Galivan et. al [18].

### 5FU functional monitoring in vitro using moAb FTS

#### Dose-response and time-dependent effects

We treated RKO and MiaPaCa2 cells with different doses (concentrations) of 5FU for 1h or 24h. By IB, RKO and MiaPaCa2 cells differed in the proportions of TS present in the TS-F form (Figure 3A). RKO cells formed TS-F at a lower dose of 5FU than did MiaPaCa2 cells. The ratio of TS-F to TS-N increased from 1h treatment to 24h treatment, as seen at the lower dose range in both cell lines. Higher 5FU doses caused more TS to be present as TS-F in both cell lines. The commercial rabbit anti-TS detected both forms of TS. MoAb FTS detected only TS-F in 5FU-treated cells. The intensity of the FTS immunoreactivity to TS-F was comparable to that of the rabbit anti-TS to TS-F. Thus, 5FU activity in cell culture was reflected in both a dose-response relationship and a time-dependence.

#### Competitions using 5FU, FUDR, and MTX

Using the rabbit anti-TS, TS-F was detected by IB after 5FU and FUDR treatments. The addition of methotrexate during treatment abrogated detection of TS-F in 5FU-treated cells and in FUDR-treated cells, by IB using the same antibody, as was shown previously [17] using TS106. The FTS antibody did not detect TS-F in the 5FU+MTX-treated samples and reacted at low intensity in FUDR+MTX-treated samples (Figure 3B). This result confirmed the known interference affecting the 5FU or FUDR modifications of TS caused by MTX [17, 19].

### 5-FU functional monitoring in vivo

#### Xenograft tissues

**Figure 4:**
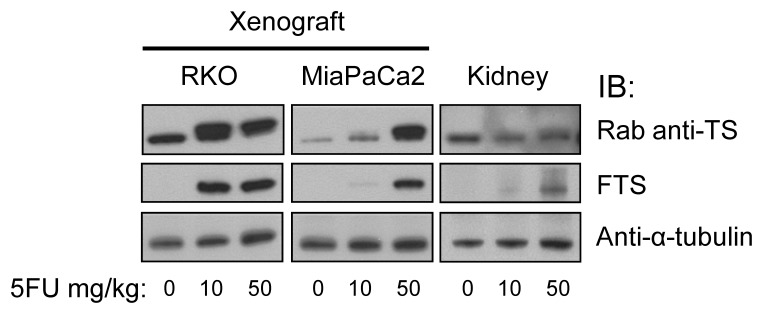
Functional monitoring of 5FU in vivo using moAb FTS Mice bearing RKO and MiaPaCa2 tumors were treated with various doses of 5FU for 1h and analyzed by IB as indicated. Rab, rabbit.

Given our proposed eventual goal of monitoring the activity of 5FU in dosed patients, we next sought to translate the demonstration to an in vivo setting. We injected athymic nude mice with RKO and MiaPaCa2 cells to develop xenograft tumors. We treated tumor-bearing mice with various doses of 5FU, harvesting tumors at one hour. By IB analysis of the tumors, we observed findings similar to those of in vitro treatments. Differences in 5FU activity were seen between the cancer lines tested. As observed in cell culture, TS-F formation in vivo was seen with lower 5FU doses in RKO than in MiaPaCa2 xenografts (Figure 4). Higher 5FU doses drove fractionally more TS conversion to the inactive TS-F form in both tumor types. The rabbit anti-TS antibody detected both forms of TS, seen as a higher TS-F:TS-N ratio with use of higher 5FU doses. MoAb FTS detected only TS-F, seen as increasing TS-F reactivity with higher 5FU doses. Thus, 5FU activity was reflected in quantitative differences paralleling a dose-response relationship and an intrinsic difference between tumors from different origins.

#### Mouse tissue and cells

From the xenograft experiments, we also harvested kidneys. TS is expected only in proliferating cells, and thus the detectable renal TS protein levels were lower than in the tumors (Figure 4). TS-F was present in kidney tissues at one hour post-treatment. FTS detected TS-F in 5FU-treated animals only. The rabbit anti-TS reacted with both TS-N and TS-F of murine kidney. The level of TS-F, and the ratio of TS-F to TS-N, were both dose-dependent. These results were replicated in an independent xenograft-treatment experiment. Separately, in 5FU-treated CT38 murine cells in culture, FTS detected TS-F alone while the rabbit anti-TS reacted with both TS-N and TS-F (Data not shown).

## DISCUSSION

A new rat monoclonal antibody, FTS, recognizes TS-F in 5FU-treated cells and tissues of human and mice. Using FTS in immunoblot assays, we observed dose-dependent responses of 5FU in cell lines and tissues. A progression of the 5FU effect was observed over the time elapsed, subsequent to dosing from 1h to 24h, RKO cells being more sensitive than MiaPaCa2 cells. Additional observations further supported the phosphate group being required for stabilization of the ternary-complex, as suggested by Brody et. al [17]. FTS detected smaller products of rTS-F after proteolytic digestion.

Although 5FU metabolites target TS, RNA, and DNA, we speculate that an immunoassay using paired and differing antibody specificities to TS might permit functional monitoring of the effectiveness of a dose of 5FU or response to 5FU in the clinic, obtaining samples by biopsy or at the time of tumor resection. Quantification based on IB, however, is tedious, requiring significant sample mass, multiple-step processing, and time. Johnston et. al used a chemiluminescence ELISA, for example, to quantify native TS in tissues using TS106 [20]. A further development, using two antibodies, one specific to TS and second specific to TS-F, might permit a liquid-based assay or other rapid automated assay to be developed for small sample size. Using the separate quantification of TS and TS-F, it would be possible to calculate a TS-F:TS-N ratio as a cumulative measure of 5FU intracellular effect over time. The FTS antibody may also aid biological experimentations of the 5FU modification of TS in vivo and in vitro, and aid comparisons of pharmacologic analogues such as 5FU, capecitabine, FdUR, and FUR.

## MATERIAL AND METHODS

### Ethics statement

Animals were treated as per institutional animal care and use guidelines. Injections were carefully done to minimize animal pain. Mice were monitored daily for health check and euthanized using carbon dioxide at the end of experiment.

### Reagents and antibodies

BL21-CodonPlus (DE3)-RIPL bacteria were purchased from Agilent Technologies. Glutathione-sepharose 4B, proteinG-sepharose column and pGEX-2TK plasmid were from GE Life Sciences. Reduced glutathione, tetrahydrofolate (THF), 5FU, methotrexate (MTX), 5-fluoro-2'-deoxyuridine (FUDR), trypsin and bovine thrombin were from Sigma-Aldrich. Calf intestinal phosphatase (CIP) and Endoproteinase AspN were from New England BioLabs. A mouse monoclonal anti-TS antibody (TS106) was purchased from Millipore, and a rabbit monoclonal anti-TS antibody, from Epitomics, Inc. Secondary horseradish peroxidase (HRP)-tagged antibodies (goat anti-rabbit IgG, goat anti-mouse IgG and chicken anti-rat IgG) were from Santa Cruz Biotechnology. Antibodies against folic acid and folinic acid (FA) were purchased from Santa Cruz Biotechnology and Abcam. Pancreatic MiaPaCa2 and colorectal RKO cancer cells were from ATCC.

### Cloning

Total RNA was isolated from RKO cells using RNeasy Mini Kit (Qiagen). cDNA was synthesized from total RNA using Superscript III cDNA synthesis kit (Invitrogen) as per manufacturer's instructions. From cDNA, the TS gene coding sequence was amplified by PCR using primers TYMS-F:5'-GTGGTGGAATTCTATGCCTGTGGCCGGCTCGGAGCTG-3' and TYMS-R:5'-CTAGACTCGAGCTAAACAGCCATTTCCATTTTAATAGT-3'. The amplified TS sequence was cloned into the GST-tagged bacterial protein expression plasmid pGEX-2TK between EcoRI and BamHI restriction enzyme sites to form pGEX-2TK-TS, the insert of which was verified by DNA sequencing.

### Isolating recombinant protein

BL21CodonPlus (DE3)-RIPL bacterial cells were transformed with pGEX-2TK-TS plasmid by heat shock. Transformed bacteria were grown in media and induced for N-terminal GST-tagged TS protein synthesis by adding isopropyl β-D-1-thiogalactopyranoside (IPTG) at 0.1mM final concentration for 3h at 37°C. The bacterial pellet was resuspended in buffer A (150mM NaCl, 50mM Tris-HCl, 1mM EDTA) with protease inhibitors (Complete, Roche), and lysozyme, DNase I, and RNase added to final concentrations of 1mg/ml, 50μg/ml, and 50μg/ml, respectively. To lyse bacteria, TritonX was added to a final concentration of 1% and the mixture vortexed and freeze-thawed thrice using liquid nitrogen. The lysate was clarified by centrifugation at 14000g for 15min. NaCl was added to the supernatant to achieve a final concentration of 0.5M, mixed well by pipetting. To isolate GST-tagged TS, glutathione sepharose 4B was added and rotated end-over-end at room temperature (RT) for 2h. The bound rGST-TS slurry was washed three times with buffer A. Recombinant GST-TS was eluted by incubating the slurry with 10mM reduced glutathione in 50mM Tris-HCl. Alternately, to elute recombinant GST-free TS (rTS), we incubated the slurry with bovine thrombin and phosphate-buffered saline (PBS) for 18hr at RT, pelleted the beads, and recovered rTS in the supernatant. We quantified protein using the D_C_ protein assay (Bio-Rad).

### In-vitro modification of rTS and TS

We modified rTS based on the molar reaction ratios: 1 part rTS + 20 parts FdUMP + 112 parts DMTHF. DMTHF was synthesized from THF as described [21], omitting the purification of DMTHF. A mixture of rTS and FdUMP incubated at RT for 1.5h. DMTHF was added, incubating at RT in the dark for 2.5h. Reagents were removed by filtration (3kD filter, Millipore) and modified rTS (rTS-F) collected. An RKO cell lysate was modified by adding FdUMP at 5μM final concentration, incubating for 30min at RT, adding DMTHF at 2.8mM final concentration, and incubating in the dark at RT for 45min.

### Antibody generation

An attempt in mice to generate a TS-F-specific monoclonal antibody was unsuccessful. rTS-F was then used to immunize rats and generate a monoclonal antibody. Hybridoma clones were screened for clone expression based on enzyme-linked immuno sorbent assay (ELISA) reactivity to rTS-F as compared to rTS (Creative Diagnostics). One clone was verified by IB to be reactive specifically to TS-F and not to TS-N upon testing TS proteins from multiple sources (see below).

### Cell line treatment

RKO and MiaPaCa2 cells were grown in 6-well plates in DMEM (10%FBS, 1% pen/strep). Cells were treated with 5FU at final concentration of 0, 0.4, 2, 10, and 50μM for 1h or 24h. At the end of the treatment, cells were lysed using detergent (1% Triton X, 150mM NaCl, 50mM Tris-HCl, 1mM EDTA) and rotated for 30min at 4°C. After pelleting by microcentrifugation for 10min, the supernatant was retained for protein studies.

In a separate experiment, RKO cells were treated with 5FU or FUDR (each at 10μM final concentration), with or without methotrexate (25μM final concentration). After 24h treatment, cells were processed as above.

### Phosphatase treatment

50μg of 5FU-treated RKO cell lysate protein was treated with 50u of CIP for 1h at 37°C. 50ng of rTS-F was treated with 2.5u of CIP for 1h at 37°C. At the end of incubation, SDS loading buffer was added and samples heated at 100°C for 5min.

### Proteolytic digestion

10μg of rTS or rTS-F was digested with trypsin or AspN as 20:1 protein:enzyme ratio for 16h at 37°C. At the end of incubation, SDS loading buffer was added and samples heated at 100°C for 5min.

### Protein gels and immunoblots

Protein gels and IB methods were as described [22]. We used 4-12% or 10% Bis-Tris pre-cast gels (Invitrogen). Coomassie staining of protein gels was done as described [23]. For IB, 30μg of protein per well was separated by elctrophoresis, gels were transferred to PVDF membrane, blocked with 5% dry non-fat milk, and probed with antibodies in 5% milk. After reaction with secondary antibodies, membranes were developed using chemiluminescence reagents (Thermo Scientific), and signal was captured using film. Each experiment was performed at least twice; representative data are shown.

### Xenograft experiments

Female athymic nude mice (Simenson Laboratories, CA) were subcutaneously injected with RKO or MiaPaCa2 cells at two flank sites. Each site was injected with one million cells in Matrigel (BD Biosciences). Mice developed xenograft tumors after three to four weeks. Tumor-bearing mice were treated with 5FU intravenously at doses of 0, 10, and 50mg/kg. Mice were euthanized using carbon dioxide at 1h after injection. Tumors were collected and snap-frozen in liquid nitrogen for storage at −80°C until analysis. The experiment was performed twice.

### Cell culture

RKO and MiaPaCa2 cells and rat hybridoma clone FTS were cultured in DMEM with 10% FBS and 1% pen/strep. FTS antibody was purified from serum-free hybridoma conditioned medium (BD Biosciences) using a ProteinG column (GE Life Sciences) as per manufacturer's protocol. Antibody was eluted with 0.1M glycine, pH 2.7, neutralized with Tris-HCl, pH 8.5, concentrated by 3kD filter, and quantified by the D_C_ protein assay and by visualization in a Coomassie-stained denaturing protein gel.
